# Both cardiovascular and non-cardiovascular comorbidity are related to health status in well-controlled type 2 diabetes patients: a cross-sectional analysis

**DOI:** 10.1186/1475-2840-11-121

**Published:** 2012-10-05

**Authors:** Paulien R Wermeling, Kees J Gorter, Henk F van Stel, Guy EHM Rutten

**Affiliations:** 1 Julius Center for Health Sciences and Primary Care, University Medical Center Utrecht, Utrecht, The Netherlands

**Keywords:** Type 2 diabetes mellitus, General practice, Health status, Comorbidity

## Abstract

**Background:**

Type 2 diabetes patients have a decreased health-related quality of life compared to healthy persons, especially regarding physical functioning and well-being. Health-related quality of life is even lower in type 2 diabetes patients when other diseases co-exist. In contrast to earlier studies, we assessed the associations between the number and type of comorbidities and health status in well-controlled type 2 diabetes patients, in whom treatment goals for HbA1c, blood pressure and cholesterol had been reached. Approximately one in five type 2 diabetes patients belongs to this group.

**Methods:**

Cross-sectional analysis was performed in 2086 well-controlled (HbA1c ≤58 mmol/mol, systolic blood pressure ≤145 mmHg, total cholesterol ≤5.2 mmol/l and not using insulin) type 2 diabetes patients in general practice. Both number and type (cardiovascular and non-cardiovascular) of comorbidities were determined for each patient. Health status was assessed with the questionnaires Short Form-36 (SF-36) and EuroQol (EQ). The SF-36 generates eight dimensions of health and a Physical and Mental Component Score (PCS and MCS), scale: 0–100. The EQ consists of two parts: EQ-5D and EQ Visual Analogue Scale. Multivariable linear regression analysis was used to assess if number and type of comorbidities were associated with health status.

**Results:**

Well-controlled type 2 diabetes patients with comorbidities had a much lower health status, with a decrease ranging from -1.5 for the MCS to -26.3 for role limitations due to physical problems, compared to those without. Health status decreased when the number of comorbidities increased, except for mental health, role limitations due to emotional problems, MCS and both EQ measures. In patients with both cardiovascular and non-cardiovascular comorbidity, physical functioning, role limitations due to physical problems and PCS were significantly lower than in patients with only cardiovascular comorbidity. Physical functioning was also lower compared to patients with only non-cardiovascular comorbidity.

**Conclusions:**

Even acceptable values of HbA1c, blood pressure and cholesterol in type 2 diabetes patients are not necessarily related with a good health status. We have shown that comorbidities have a large impact on health status. Physicians may take into account patient’s health status and integrate the impact of comorbidities into diabetes care.

## Background

Type 2 diabetes patients have a decreased health-related quality of life compared to healthy persons, especially regarding physical functioning and well-being [1,2]. Furthermore, diabetes patients with co-existing macrovascular or non-vascular diseases have an even lower health-related quality of life [2].

Cardiovascular diseases [3,4] are associated with a lower health-related quality of life but also more specific types, like: myocardial infarction [5], stroke [5-8], heart disease [7-9], heart failure [5,10] and peripheral vascular disease [7]. Non-vascular diseases which are associated with a lower health-related quality of life are: emphysema/COPD [3,4], (osteo)arthritis [3,8,9,11] and depression [5,6,8,10,12]. For most of these comorbidities mainly physical health was decreased, except for depression.

Not only the type of comorbidity but also the absolute number of diseases is associated with a decreased health-related quality of life in type 2 diabetes patients [7,8,11,13,14]. Type 2 diabetes patients with comorbidities visit their general practitioner and medical specialist more often and are more frequently admitted to the hospital than patients without [15]. Therefore, this group deserves special attention.

In contrast to the above mentioned research, we studied the health status of well-controlled type 2 diabetes patients, in whom treatment goals for HbA1c, blood pressure and cholesterol had been reached. About one in five type 2 diabetes patients belongs to this group [16,17]. Many physicians are likely to be satisfied if treatment goals are achieved, but also health status should be addressed according to the American Diabetes Association [18]. Therefore, we aimed to assess the association between the number and type of comorbidities and health status in a large sample of well-controlled type 2 diabetes patients in general practice.

## Methods

### Design

We present a cross-sectional analysis on baseline data from the EFFIMODI study. The design and rationale of EFFIMODI have been described elsewhere [19]. Patients were selected from the computerised medical records of 233 general practitioners across the Netherlands. Patients were eligible if between 40 and 80 years old, diagnosed with type 2 diabetes for more than a year, treated by their general practitioner, not on insulin treatment and were well-controlled, defined as having HbA1c ≤58 mmol/mol (≤7.5%), systolic blood pressure ≤145 mmHg and total cholesterol ≤5.2 mmol/l. These cut-off values were a little higher than the Dutch target values, namely HbA1c ≤53 mmol/mol (≤7.0%), systolic blood pressure ≤140 mmHg and total cholesterol ≤4.5 mmol/l [20].

The general practitioner sent an information letter as well as an informed consent form to all eligible patients by mail. Patient who were willing to participate signed informed consent. After returning the informed consent form, they received a postal questionnaire to complete and return it to the investigators. In case of non-response they received two reminders.

The Medical Research Ethics Committee of the University Medical Center Utrecht has approved the study protocol (Protocol number: 08–453) and we conducted the study according to the 1964 Declaration of Helsinki.

### Measurements

Both the patient questionnaire and medical data were used. Medical data of the patients were collected by a case report form that was filled in by the general practitioner or practice nurse. Data were collected about the following eleven conditions: myocardial infarction, angina pectoris, heart surgery, heart failure, stroke, transient ischemic attack, peripheral arterial disease, COPD, rheumatoid arthritis, osteoarthritis of hip or knee or any other diseases.

Despite the fact that the terms ‘health status’ and ‘health-related quality of life’ have different meanings, they are used interchangeably in literature. Impaired health status may lead to impaired quality of life, but this is not always the case [21]. We have chosen to measure health status, because this is relatively easy to measure in daily practice [22].

Health status was measured with the self-administered questionnaires Short Form-36 (SF-36) [22] and EuroQol [23,24]. The Dutch translation of the SF-36 has been validated in both general and disease-specific samples [25]. The SF-36 consists of 36 questions and generates a profile of scores on eight dimensions of health, namely: physical functioning (PF), role limitations due to physical problems (RP), bodily pain (BP), social functioning (SF), mental health (MH), role limitations due to emotional problems (RE), vitality (VT) and general health (GH). For all eight dimensions a score is calculated, with a range from 0 (least favourable health state) to 100 (most favourable health state) [26]. Two summary scales with a similar range for mental and physical functioning can be calculated as well: the Physical Component Score (PCS) and the Mental Component Score (MCS) [27].

The EuroQol (EQ) is a generic questionnaire, consisting of a classification system (EQ-5D) and a Visual Analogue Scale (EQ VAS). The EQ-5D covers five dimensions of health (mobility, self-care, usual activities, pain/discomfort and anxiety/depression), each with three levels of functioning: no problems, some problems and severe problems. These questions were used to compute an index value based on a Dutch valuation study [28]. The index value ranges between +1 and −0.329, where 0 means death. The EQ VAS is a graded, vertical line, anchored at 0 (worst imaginable health state) and 100 (best imaginable health state). The patient is asked to mark a point on the EQ VAS that best reflects his/her actual health state.

Age, gender, ethnicity (Dutch and non-Dutch), educational level (low, middle or high) and living alone (yes or no) were collected with the patient questionnaire and BMI (in kg/m^2^) and duration of diabetes (in years) were collected with the case report form. We considered these variables as potential confounders based on literature [1,3,7,9,29]. HbA1c, blood pressure and lipid levels were not considered as potential confounders because the well-controlled study population was rather homogeneous in this respect. Also, because of the low levels of these variables, they were not likely to be associated with health status.

### Statistical analysis

Descriptive statistics were used to describe the characteristics of the population. Categorical variables were expressed as percentages and continuous variables as a mean with standard deviation (SD). From the answers to the open question of ‘any other diseases’, we selected ‘cancer of all causes’ (except skin cancers other than melanoma) to be added to the comorbidities because of its frequently mentioned occurrence.

Patients were categorised into those with no, one, two and more than two comorbidities. Every specific condition was counted as one comorbidity, except having a stroke or transient ischemic attack. Having one or both of these conditions was counted as one comorbidity. Myocardial infarction, angina pectoris, heart surgery, heart failure, stroke, transient ischemic attack and peripheral arterial disease were aggregated as cardiovascular comorbidities; and all other conditions as non-cardiovascular.

To assess the relation between comorbidity and health status we used: the eight health status dimensions of the SF-36, the PCS and the MCS of the SF-36, the EQ-5D and the EQ VAS. Comorbidity was categorised in two different ways: 1) no, one, two and more than two comorbidities and 2) diabetes only, diabetes with cardiovascular comorbidity, diabetes with non-cardiovascular comorbidity and diabetes with both cardiovascular and non-cardiovascular comorbidities. For each health status measure a multiple linear regression model was used to assess the association between type and number of comorbidities and health status, adjusted for potential confounders. These linear regression models give Betas for each group of comorbidity. The Betas are the differences in health status compared to the reference group (no comorbidity or diabetes only), for example a Beta of −5 means that a group scored 5 points lower on a particular health status score than the reference group. A p-value of <0.05 was considered statistically significant. Although health status had a skewed distribution in our study population, we chose to perform a linear regression analysis, to control for confounders. The latter is not possible with Mann–Whitney or Kruskal-Wallis tests.

For handling missing data in the confounders and health status domains we used multiple imputation. We assumed that the missing data were missing at random. We generated 10 imputed datasets and used Rubin’s rules to combine the estimates of the parameters [30].

To examine if significantly different health status scores between groups of comorbidities are clinically relevant, we used the commonly used effect size: the average difference between groups divided by the SD [31]. An effect size of >0.2 is called a small effect, >0.5 is a medium effect and >0.8 is a large effect [31]. Data were analysed using SPSS version 17 and R version 2.10.

## Results

The study population consisted of 2215 type 2 diabetes patients. We excluded patients that did not fill in a questionnaire (n=96), with missing medical data (n=28) or who missed both (n=5), leaving 2086 patients for further analyses. Their mean age was 65 years, 60% were males and the mean diabetes duration was 6 years. 62% of the type 2 diabetes patients had no comorbidity, 24% had one comorbidity, 9% had two comorbidities and 5% had more comorbidities. Of all patients, 26% had a cardiovascular comorbidity and 18% a non-cardiovascular comorbidity (Table 1).

**Table 1:** Characteristics of the study population (N=2086)

	**n**	
Age (years)	2086	64.4 ± 8.8
Gender (male)	2086	1240 (59.4%)
Dutch ethnicity	2073	1852 (89.3%)
Educational level	1912	
Low		789 (41.3%)
Middle		699 (36.6%)
High		424 (22.2%)
Living alone	2053	399 (19.4%)
BMI (kg/m^2^)	2039	29.2 ± 4.7
Duration of diabetes in years	2034	5.8 ± 3.7
No comorbidity	2086	1290 (61.8%)
One comorbidity	2086	517 (24.8%)
Two comorbidities	2086	179 (8.6%)
More than two comorbidities	2086	100 (4.8%)
Cardiovascular comorbidity	2086	549 (26.3%)
Non-cardiovascular comorbidity	2086	383 (18.4%)

Data are means **±** SD or n (%).

Type 2 diabetes patients with comorbidities had a much lower health status compared to those without any comorbidity. Especially type 2 diabetes patients with co-existing heart failure, peripheral arterial disease, COPD and rheumatoid arthritis had a lower health status (Table 2).

**Table 2:** Mean health status scores for type 2 diabetes patients with and without different types of comorbidity

	**Diabetes only n=1290**	**Myocardial infarction n=180**	**Angina pectoris n=200**	**Heart surgery n=118**	**Heart failure n=57**	**Stroke n=120**	**Transient ischemic attack n=54**	**Peripheral arterial disease n=96**	**COPD n=147**	**Rheuma-toid arthritis n=40**	**Osteo-arthritis hip/knee n=176**	**Cancer n=69**
**PF**	81.4	69.7	69.7	64.0	56.5	67.7	71.3	59.2	63.0	59.4	61.2	66.6
**RP**	82.1	70.7	70.4	56.9	61.6	73.0	74.8	67.1	65.5	53.4	66.3	74.6
**BP**	79.8	74.6	73.2	68.6	72.8	75.5	79.7	68.3	74.1	62.3	66.3	73.6
**SF**	87.2	80.2	83.3	78.0	74.1	77.7	80.5	80.4	81.5	78.2	78.7	77.6
**MH**	80.7	77.3	78.0	77.9	73.9	75.7	79.1	76.7	76.9	78.1	77.7	75.3
**RE**	90.1	84.3	83.9	83.8	73.8	84.0	82.3	84.2	84.1	82.9	84.0	83.9
**VT**	69.3	64.2	64.8	61.3	58.6	62.0	64.9	62.0	60.3	59.6	63.3	61.8
**GH**	66.3	58.4	57.6	52.9	52.0	59.1	63.0	58.0	52.8	52.4	58.7	59.7
**PCS**	48.3	44.3	43.8	40.1	41.2	44.6	45.6	41.3	41.9	38.0	41.2	45.3
**MCS**	54.4	53.2	53.8	53.5	51.5	52.2	52.8	54.3	53.7	54.8	54.6	52.7
**EQ-5D**	0.88	0.84	0.84	0.82	0.81	0.80	0.82	0.77	0.82	0.76	0.77	0.81
**EQ VAS**	78.7	74.0	72.8	71.4	69.4	73.1	75.1	70.1	69.7	69.6	72.5	71.1

Data are means.

COPD, Chronic Obstructive Pulmonary Disease; PF, physical functioning; RP, role limitations due to physical problems; BP, bodily pain; SF, social functioning; MH, mental health; RE, role limitations due to emotional problems; VT, vitality; GH, general health; PCS, Physical Component Score; MCS, Mental Component Score; EQ-5D, EuroQol 5 Dimensions; EQ VAS, EuroQol Visual Analogue Scale.

The N does not sum up to the total number of the study population. This is because when a patient has two or more comorbidities, it will be handled into two or more of the groups.

All health status domains were significantly lower in patients who had one or more comorbidities compared to type 2 diabetes patients with no comorbidities (Table 3). Almost all health status measures further decreased when the number of comorbidities increased, with the exception of mental health, role limitations due to emotional problems, mental component score, EQ-5D and EQ VAS. Physical functioning and general health showed a significant decrease (−5.5 and −5.2, respectively) between the groups with one and with two comorbidities. The health status domains physical functioning, role limitations due to physical problems, bodily pain, social functioning, vitality, general health and the physical component score differed significantly between the groups with one and with more than two comorbidities. Furthermore, the domains physical functioning, role limitations due to physical problems and the physical component score were significantly lower (-8.0, -14.8 and -3.8, respectively) in the group with more than two comorbidities compared to the group with two comorbidities.

**Table 3:** Number of comorbidities and difference in health status domains compared to people with 0 comorbidities

	**0 comorbidity n=1290**	**1 comorbidity n=517**	**2 comorbidities n=179**	**>2 comorbidities n=100**
	**Mean ± SD**	**Beta (95% CI)**	**Beta (95% CI)**	**Beta (95% CI)**
PF (Physical Functioning)	81.4 ± 20.3	−8.8 (−10.9 to −6.7)*	−14.3 (−17.6 to −11.0)†	−22.3 (−26.6 to −18.0)‡
RP (Role limitations due to Physical problems)	82.1 ± 33.1	−8.2 (−11.8 to −4.5)*	−11.5 (−17.1 to −5.9)*	−26.3 (−33.6 to −19.0)‡
BP (Bodily Pain)	79.8 ± 22.0	−5.9 (−8.2 to −3.5)*	−8.0 (−11.6 to −4.4)*	−13.1 (−17.7 to −8.4)†
SF (Social Functioning)	87.2 ± 18.6	−5.0 (−7.1 to −3.0)*	−7.5 (−10.6 to −4.3)*	−12.0 (−16.1 to −7.9)†
MH (Mental Health)	80.7 ± 15.0	−3.6 (−5.2 to −2.0)*	−4.5 (−6.9 to −2.1)*	−4.6 (−7.8 to −1.5)*
RE (Role limitations due to Emotional problems)	90.1 ± 26.0	−6.0 (−9.1 to −2.9)*	−7.3 (−12.0 to −2.5)*	−10.6 (−16.7 to −4.4)*
VT (Vitality)	69.3 ± 17.7	−5.5 (−7.3 to −3.7)*	−8.6 (−11.4 to −5.8)*	−11.7 (−15.3 to −8.0)†
GH (General Health)	66.3 ± 18.6	−6.1 (−8.1 to −4.2)*	−11.3 (−14.2 to −8.3)†	−13.3 (−17.2 to −9.5)†
PCS (Physical Component Score)	48.3 ± 9.4	−3.3 (−4.3 to −2.3)*	−5.4 (−6.9 to −3.9)*	−9.2 (−11.2 to −7.3)‡
MCS (Mental Component Score)	54.4 ± 8.2	−1.5 (−2.4 to −0.6)*	−1.8 (−3.1 to −0.4)*	−1.6(−3.3 to 0.2)
EQ-5D (EuroQol 5 Dimensions)	0.88 ± 0.16	−0.06 (−0.08 to −0.05)*	−0.07 (−0.10 to −0.05)*	−0.11 (−0.15 to −0.08)*
EQ VAS (EuroQol Visual Analogue Scale)	78.7 ± 13.8	−4.8 (−6.3 to −3.4)*	−7.8 (−10.0 to −5.5)*	−8.9 (−11.8 to −6.0)*

Linear regression analyses were adjusted for age, gender, ethnicity, education, living alone, BMI and duration of diabetes.

* significantly different from the group with 0 comorbidity.

† significantly different from the group with 0 and the group with 1 comorbidity.

‡ significantly different from the groups with 0, 1 and 2 comorbidities.

SD, standard deviation; 95% CI, 95% confidence interval.

Patients with only diabetes had a significantly higher health status on all domains (Table 4). Whether patients had a cardiovascular or non-cardiovascular comorbidity was not significantly associated with their health status. If patients had both cardiovascular and non-cardiovascular comorbidities, they had a significantly lower score (-6.8 compared to only non-cardiovascular and -9.0 compared to only cardiovascular) on physical functioning. Compared with patients with only cardiovascular comorbidity, patients with both cardiovascular and non-cardiovascular comorbidity scored worse on role limitations due to physical problems and physical component score (-11.9 and -4.0, respectively). Furthermore, all other health status domains were not significantly different between the three groups.

**Table 4:** Type of comorbidity and difference in health status domains compared to people with only diabetes

	**Only diabetes n=1290**	**Diabetes with only cardiovascular disease n=413**	**Diabetes with only non-cardiovascular disease n=247**	**Diabetes with both cardiovascular and non-cardiovascular diseases n=136**
	**Mean ± SD**	**Beta (95% CI)**	**Beta (95% CI)**	**Beta (95% CI)**
PF (Physical Functioning)	81.4 ± 20.3	−9.4 (−11.8 to −7.0)*	−11.6 (−14.5 to −8.8)*	−18.4 (−22.2 to −14.6)†
RP (Role limitations due to Physical problems)	82.1 ± 33.1	−7.9 (−11.9 to −3.9)*	−11.6 (−16.6 to −6.6)*	−19.8 (−26.2 to −13.4)‡
BP (Bodily Pain)	79.8 ± 22.0	−6.0 (−8.6 to −3.4)*	−7.4 (−10.5 to −4.3)*	−10.4 (−14.5 to −6.3)*
SF (Social Functioning)	87.2 ± 18.6	−6.0 (−8.3 to −3.8)*	−5.1 (−7.8 to −2.4)*	−10.0 (−13.6 to −6.4)*
MH (Mental Health)	80.7 ± 15.0	−4.4 (−6.1 to −2.6)*	−3.1 (−5.2 to −1.1)*	−4.0 (−6.8 to −1.2)*
RE (Role limitations due to Emotional problems)	90.1 ± 26.0	−7.4 (−10.8 to −4.0)*	−4.0 (−8.1 to 0.1)	−10.6 (−16.0 to −5.2)*
VT (Vitality)	69.3 ± 17.7	−6.5 (−8.5 to −4.5)*	−6.2 (−8.6 to −3.8)*	−9.6 (−12.8 to −6.4)*
GH (General Health)	66.3 ± 18.6	−6.8 (−9.0 to −4.7)*	−8.3 (−10.9 to −5.7)*	−11.7 (−15.0 to −8.3)*
PCS (Physical Component Score)	48.3 ± 9.4	−3.3 (−4.4 to −2.2)*	−4.8 (−6.1 to −3.5)*	−7.3 (−9.0 to −5.6)‡
MCS (Mental Component Score)	54.4 ± 8.2	−2.1 (−3.0 to −1.1)*	−0.7 (−1.9 to 0.4)	−1.7 (−3.2 to −0.1)*
EQ-5D (EuroQol 5 Dimensions)	0.88 ± 0.16	−0.06 (−0.08 to −0.04)*	−0.07 (−0.10 to −0.05)*	−0.10 (−0.13 to −0.07)*
EQ VAS (EuroQol Visual Analogue Scale)	78.7 ± 13.8	−5.4 (−7.0 to −3.7)*	−5.9 (−8.0 to −3.9)*	−7.8 (−10.4 to −5.3)*

Linear regression analyses were adjusted for age, gender, ethnicity, education, living alone, BMI and duration of diabetes.

* significantly different from the group with only diabetes.

† significantly different from the other three groups.

‡ significantly different from the groups with only diabetes and diabetes with only cardiovascular disease.

SD, standard deviation; 95% CI, 95% confidence interval.

When comparing the differences between the groups on clinical importance, most domains showed a small effect (>0.2), some showed a medium effect (>0.5) (mainly physical domains) and three had a large effect (>0.8), namely zero versus more than two comorbidities on both physical functioning and the physical component score and diabetes only versus diabetes with both cardiovascular and non-cardiovascular comorbidity with respect to physical functioning.

## Discussion

This study showed that type 2 diabetes patients with a comorbidity had a lower health status than patients without. Furthermore, health status decreased with an increasing number of comorbidities, except for the mental health measures. Patients with both cardiovascular and non-cardiovascular comorbidities, had a significantly lower physical health than patients with only cardiovascular or non-cardiovascular comorbidity. The percentage of type 2 diabetes patients with one or more comorbidities in our study was 38%, while in another study in the Netherlands this was 44% [15]. This difference is probably due to our selection of well-controlled, no insulin using diabetes patients.

While the link between diabetes patients with comorbid diseases and poor health status is well established, our study is novel because it looked at a group of people with well-controlled type 2 diabetes. Our results are consistent with findings from general diabetes populations. We found that comorbidity in diabetes patients was mainly associated with diminished physical health, as was described for osteoarthritis, stroke, cardiovascular disease, respiratory disease and myocardial infarction [3-7,11]. Furthermore, we also found that a higher number of comorbidities is associated with a decreased health-related quality of life in type 2 diabetes patients [7,8,11,13,14].

In contrast to aforementioned types of comorbidities, comorbid depression is associated with both a lower physical and mental health status [5,6,8,10,12]. This means that the mental health status scores are likely to be associated with depression, however we did not measure depression. We found that a higher number of comorbidities did not further decrease mental health, role limitations due to emotional problems and mental component score. Furthermore, the Mental Component Scores in our population were rather high (mean: 54) indeed and patients with scores above 42 are not likely to have a depression [26]. These findings suggest that depression (and thus a decreased mental health) was not a major issue in our study population. This is in accordance with the fact that depression is associated with poor glycemic control [32] and we studied a selection of well-controlled diabetes patients.

The decrease in health status with increasing number of comorbidities seen in most SF-36 domains was also seen in the EQ-5D and EQ VAS. However, these changes were not significant. This could be explained by the fact that these health status measures combine both physical and mental health in one score. Therefore, the differences between one, two and more than two comorbidities might be diluted by the large differences for physical health on one side and almost no differences for mental health on the other. Furthermore, this could be due to the relatively small groups of people with two and more than two comorbidities. Also, we found rather high values for health status. This could be due to the fact that poor self-rated health is associated with increasing glucometabolic disturbances [33] and such patients were not included in the study.

Also patients with diabetes and microvascular complications [7,10,34,35] have a lower health-related quality of life compared to patients with diabetes alone; however we did not look at the association between microvascular complications and health status. Patients with end stage renal disease were not included, because they are not treated by their general practitioner. The same applies to patients with diabetic foot disease or neuropathy. Retinopathy is estimated to be present in about 7.4% of the type 2 diabetes patients in the Netherlands [36]. Because our population had a relatively short diabetes duration and people were well-controlled, our retinopathy prevalence would have been low. This was confirmed by the fact that retinopathy was only mentioned nine times among ‘other diseases’. Therefore we think this will not have influenced our results.

Several studies studied the relation between comorbidity and glycemic control and since we looked at comorbidity in a selection of well-controlled type 2 diabetes patients, their findings might be of interest to interpret our findings. The risk of cardiovascular diseases increases with a higher HbA1c [37], so our study population had a relatively low risk of getting ‘new’ complications. On the other hand, diabetes patients with coronary heart disease or congestive heart failure have a lower odds of having at least one cardiovascular risk factor (glycemia, blood pressure and lipids) out of control [38]. Since our results are similar to studies in general (both well-controlled and not well-controlled patients) diabetes populations, we think that ‘good cardiometabolic control’ was not a confounder in the association between comorbidity and health status. In the relationship between diabetes control and comorbidities, general practitioners’ treatment and patients’ health behavior are likely to play a role as well [39]. Because of the homogeneity of the study population (all were well-controlled) we were not in the position to elucidate whether the health status of patients is associated with patient’s behavior.

Although quality of care may increase with an increasing number of chronic conditions [40], our study demonstrated that acceptable or good cardiometabolic control does not automatically reflect a good health status. So far, three types of interventions were designed to improve quality of life in diabetes patients: a disease management program [41], implementing several elements of the Chronic Care Model [42] and a structured group self-management educational intervention [43]. Diabetes patients in the German disease management program had higher health-related quality of life in the dimensions mobility, self-care and performing usual activities compared to routine care. The same was shown for patients with a high number of comorbidities [41]. The disease management program also improved processes of care and intermediate outcomes. However, there were no differences in intermediate outcomes between the disease management program and routine care [44]. The number of secondary diseases and the presence of a disabling secondary disease were related to drop-out of the disease management program [45]. This disease management program showed that it may improve health-related quality of life and therefore may be useful in clinical practice. However, one might question their usefulness for diabetes patients with comorbidities. Disease management programs focus on a single disease and as we could demonstrate health status does not depend on a single disease. In our opinion the above mentioned drop-out is not surprising; in disease management programs special attention should be paid to patients with both cardiovascular and non-cardiovascular comorbidity. The other two mentioned interventions are still ongoing.

Strengths of this study are that we have measured multiple health status domains and multiple comorbidities. On top of that, we have measured them all in one, large population. This allowed us to quantify the impact of comorbidity on several health status domains. This could be helpful in providing specific treatment options to type 2 diabetes patients, depending on the different comorbidities and the different aspects of their health status. Because the included patients were not only recruited for a randomised controlled equivalence trial but were also part of a patient preference study, we minimised selection bias [46]. Besides, we had a high response rate on the patient questionnaires, probably due to the fact that patients had already agreed to participate in a larger study and thus were more motivated to fill in the questionnaire.

There are also limitations that need to be addressed. Firstly, comorbidity in our study population might be underreported, and lack of disease coding in the electronic medical records might play a role. We cannot assess its role, but underreporting is not likely to influence the direction of our results. Secondly, we selected only eleven conditions, seven of which were vascular diseases. Conceptually, multimorbidity includes all potential other conditions. However, other diseases were hardly reported as meaningful diseases, with the exception of cancer. Therefore we assume that this will not have biased our results. Thirdly, we have no information about the medication used for the comorbidity. So we do not know if it is the comorbidity itself that decreased health status or the medication patients are using for it. Fourthly, we wanted to assess the impact of comorbidity regardless of possible confounders such as age, gender, ethnicity and educational level of patients. However, adjusting for these possible confounders implies that the impact of these confounders on health status could not be assessed separately. Lastly, because of the cross-sectional design no causal relationship could be established. However, a cohort study with a follow-up of five years showed that in elderly diabetes patients the diabetes-related complications were predictive of reduced health-related quality of life, but the number of comorbid diseases did not [47]. We found that both diabetes-related complications (cardiovascular comorbidity) and the number of comorbidities were associated with a reduced health status, so the cohort study only partly confirms to our results. The fact that in the cohort study the number of comorbid diseases did not predict reduced health-related quality of life may be explained by the fact that at baseline health-related quality of life was already lower in diabetes patients with comorbid diseases, therefore during the study period no further reduction in health-related quality of life might be detectable.

## Conclusions

Our study demonstrates that even acceptable values of HbA1c, blood pressure and cholesterol in type 2 diabetes patients are not enough for good clinical care. A higher number and both cardiovascular and non-cardiovascular comorbidities are associated with a decreased health status. Increasing the health status of type 2 diabetes patients is necessary, irrespective of comorbidity, and should be considered a physician’s task. Our data suggest that physicians should not be satisfied if a patient with type 2 diabetes has achieved acceptable values of HbA1c, blood pressure and cholesterol, but may take into account patient’s health status and integrate the impact of comorbidities into diabetes care.

## Competing interests

The authors declare that they have no competing interests.

## Authors’ contributions

GEHMR is the principal investigator of the study. PRW collected and analysed the data. HFvS participated in data analysis. PRW drafted the manuscript which was critically revised by KJG, HFvS and GEHMR. All authors read and approved the final manuscript.
